# Mechanical Modification of Poly(3-hydroxybutyrate-co-3-hydroxyhexanoate) by Addition of Crosslinked Poly(3-hydroxybutyrate-co-3-hydroxyhexanoate) Particles

**DOI:** 10.3390/polym17101318

**Published:** 2025-05-12

**Authors:** Arisa Sonoyama, Masayuki Yamaguchi

**Affiliations:** 1 Kaneka Corporation, 5-1-1 Torikai-Nishi, Settsu 566-0072, Osaka, Japan; 2 Graduate School of Advanced Science and Technology, Japan Advanced Institute of Science and Technology, Nomi 923-1292, Ishikawa, Japan

**Keywords:** poly(3-hydroxybutyrate-co-3-hydroxyhexanoate), impact strength, fracture mechanism, biodegradation

## Abstract

In the present study, we prepared crosslinked particles of poly(3-hydroxybutyrate-co-3-hydroxyhexanoate) (PHBH) to investigate their performance as impact modifiers. The mechanical properties of crystalline PHBH comprising 5.6 mol% 3-hydroxyhexanoate (3HHx) were modified by the addition of amorphous particles of PHBH with 28 mol% 3HHx (PHBH28). The tensile impact strength of the mixture was improved by the addition of PHBH28 particles, particularly when they were crosslinked. The size of the dispersed uncrosslinked PHBH28 particles was sensitive to the processing method. However, the crosslinked PHBH28 particles retained their form under any processing conditions, and a smaller particle size was achieved, leading to shear yielding. The samples containing crosslinked PHBH28 particles exhibited intense stress-whitening after impact testing. The resulting voids were ascribed to cavitation in the particles, which must have been responsible for the improved mechanical properties of the samples. Moreover, the crosslinked particles did not affect the excellent biodegradability of PHBH in seawater.

## 1. Introduction

The increasing interest in biodegradable bioplastics stems from the urgent need to address environmental pollution caused by traditional plastic materials [1]. These environmentally friendly alternatives offer promising solutions to reduce the detrimental effects of plastic waste accumulation on ecosystems and human health. Utilizing biodegradable bioplastics is a vital approach to fostering sustainability and conserving natural resources [2,3,4].

Polyhydroxyalkanoates (PHAs) are biodegradable bioplastics that have garnered significant attention. They are bio-derived aliphatic polyesters that accumulate within microbial cells as energy reserves [5,6,7,8]. PHAs are readily biodegradable through enzymatic and hydrolytic processes in both marine and soil environments [9,10,11,12].

PHAs encompass a range of highly chiral homopolymers, such as poly[(*R*)-3-hydroxybutyrate (3HB)] (PHB), as well as copolymers with varying alkyl side chain lengths. The properties of PHAs are influenced by their copolymer composition [13,14,15]. For instance, PHB is known for its high crystallinity and brittleness, whereas copolymers with longer alkyl side chains are more flexible. A copolymer of 3HB and 3-hydroxyhexanoate (3HHx), i.e., poly(3-hydroxybutyrate-co-3-hydroxyhexanoate) (PHBH), which is a commercially available marine-degradable PHA (Green Planet^TM^) produced by Kaneka Corporation (Osaka, Japan), is a notable example.

Although PHAs have significant potential as alternatives to traditional fossil fuel-based plastics, their usefulness is constrained by their poor mechanical properties [16,17,18]. This is ascribed to the relatively high glass transition temperature [19] and a low density of entanglement couplings, i.e., a high entanglement molecular weight [20]. The blending of PHAs with other materials has been investigated to overcome this problem. The common approach is blending with another biopolymer with a low glass transition temperature, such as polycaprolactone (PCL) [21], poly(butylene succinate-co-adipate) (PBSA) [22], and poly(butylene adipate-co-terephthalate) (PBAT) [23]. The addition of ductile PHAs with no/low crystallinity has also been investigated [16,24].

It is generally known that adding rubber particles is an effective means of toughening polymers by various mechanisms such as a decrease in the dilatational stress or volumetric strain by cavitation in rubber particles, shear yielding deformations of a matrix localized between rubber particles, and multiple crazing from rubber particles [25,26,27,28,29,30]. This technique also applies to the modification of PHAs [31,32], which will avoid a brittle fracture. In many cases, however, the mechanism of toughness improvement was not studied in detail at least for PHAs containing rubber materials. Although such rubbery materials improve the mechanical properties of a plastic, with the exception of a rubbery PHA impact modifier, the biodegradability of the plastic must be adversely affected by the rubber, especially in a marine environment. Even in the case of blends with a rubbery PHA, a large amount of the impact modifier is required to improve the mechanical toughness of the plastic. This leads to problems such as a decrease in the tensile modulus and tensile strength of the plastic due to its low crystallinity [16,24]. Given these circumstances, a new approach that does not inhibit the biodegradability of PHAs and minimizes the loss of stiffness at a lower dosage is required to address the issue of current PHA blend systems and expand the application of PHAs. One good approach is to add a similar material in chemical structures to obtain fine dispersion.

It was reported that two PHBH materials with a difference in the 3HHx content of more than 20 mol% are immiscible [19]. These two PHBH materials, however, should exhibit a low interfacial tension, irrespective of the crosslinking structure. Therefore, in the present study, we chose an amorphous PHBH containing 28 mol% 3HHx (PHBH28) as the modifier owing to its favorable biodegradability and elastomeric behavior. We prepared crosslinked PHBH28 particles because crosslinked rubber is used in industry as a mechanical modifier [28,30]. The modification of the mechanical properties of crystalline PHBH following the addition of crosslinked or uncrosslinked PHBH28 was evaluated by structure characterization.

## 2. Materials and Methods

### 2.1. Materials

We used two types of PHBH samples with different 3HHx contents, both supplied by Kaneka Corporation (Osaka, Japan), in the present study. PHBH6 contained 5.6 mol% 3HHx and PHBH28 had 28 mol% 3HHx. The number- and weight-average molecular weights, evaluated by size-exclusion chromatography with a polystyrene standard, were *M_n_* = 3.4 × 10^5^ and *M_w_* = 5.6 × 10^5^ for PHBH6 and *M_n_* = 3.7 × 10^5^ and *M_w_* = 8.0 × 10^5^ for PHBH28, respectively. We evaluated glass transition temperatures by differential scanning calorimetry (DSC) at a heating rate of 10 °C min^−1^. They were 3.5 °C for PHBH6 and −7.5 °C for PHBH28. The DSC measurements revealed that PHBH28 had no crystals.

Commercially available pentaerythritol and behenamide were used without further purification.

### 2.2. Sample Preparation

#### 2.2.1. Preparation of Crosslinked PHBH28

Crosslinked PHBH28 particles, denoted as xPHBH28, were prepared using uncrosslinked PHBH28, which is denoted as uPHBH28 hereafter. The preparation method was described in detail in the previous report [33]. The volume mean diameter, which was measured with a particle size analyzer (Microtrac MT3300 EXII; MicrotracBEL Corp., Osaka, Japan) using a wet method, was 1.7 µm. The gel fraction in xPHBH28 was determined by the following method. A chloroform solution with a concentration of 0.7 wt% of xPHBH28 was prepared at 60 °C and stirred for 30 min and then allowed to cool to 23 °C. The solution was then filtered through a membrane with a pore diameter of 0.45 µm. During the filtration process, the interior of the container was rinsed several times with chloroform to ensure thorough washing. When the gel remaining on the filter had dried, its weight was determined to calculate the gel fraction. The gel fraction of the xPHBH28 used in the present study was 96%.

#### 2.2.2. Preparation of Blend Samples

PHBH6 and uPHBH28 or xPHBH28 were blended in various ratios using a co-rotating twin-screw micro-compounder with a capacity of 5 mL (Xplore MC5; Xplore Instruments B.V., Sittard, The Netherlands) with 1 part per hundred rubber (phr) of pentaerythritol and 0.5 phr of behenamide. The screws were non-notched, conical-shaped, and intermeshing. All heating zones were controlled at 175 °C to prevent thermal degradation [34]. After mixing for 3 min at a screw rotation speed of 100 rpm, the mixture was extruded through a circular die. The strands were compressed at 165 °C with a slight pressure for 6 min, subjected to 5 MPa of pressure for 2 min, then quenched at 30 °C for 2 min under 5 MPa to prepare test specimens with a given thickness. The specimens were annealed at 23 °C and 50% relative humidity (RH) for 7 days before the measurements were obtained.

### 2.3. Measurements

The temperature dependence of the dynamic tensile modulus was evaluated from −40 to 130 °C using a dynamic mechanical analyzer (DVA-200; IT Keisoku Seigyo Co., Ltd., Osaka, Japan). The measurements were obtained at a frequency of 1 Hz and a constant heating rate of 5 °C min^−1^. Rectangular samples cut from the compression-molded films (5 mm wide, 25 mm long, and 0.5 mm thick) were used for the measurements.

We examined the morphology of each sample by scanning probe microscopy (SPM) (Dimension Icon; Bruker Corp., Billerica, MA, USA) in PeakForce quantitative nanomechanical (PF-QNM^®^) mode. Prior to the investigation, each sample was sliced using an ultramicrotome (FC7; Leica Microsystems GmbH, Wetzlar, Germany) equipped with a diamond knife at −120 °C. A silicon probe (R-TESPA-300; Bruker Corp.) with a spring constant of 40 N m^−1^ was used. The strands extruded from the compounder were cut perpendicular to the flow direction. The centers of the cut surfaces were then examined. Modulus mapping and adhesion mapping images were obtained.

Thermal analysis was performed by DSC (DSC 214 Polyma; NETZSCH Group, Selb/Bavaria, Germany) under a nitrogen atmosphere. Each sample (approximately 3 mg) was placed in an aluminum pan and heated from −40 to 180 °C at a rate of 10 °C min^−1^. The crystallinity of each sample per unit weight (χ_c_) was calculated using the following equation:

χ_c_ = *Δh_F_*/*Δh_F_*^0^ × 100 (%) (1)

where *Δh_F_* is the heat of fusion of the sample and *Δh_F_*^0^ is that of the perfect crystal, i.e., 146 J g^−1^ [35].

The tensile properties of each sample film (0.2 mm thick) were measured at 23 °C using a tensile tester (EZ-LX 1 kN; Shimadzu Corp., Kyoto, Japan) according to JIS K 7127. The test speed was 100 mm min^−1^, and the initial distance between the clamps was 40 mm. The measurements were performed five times for each sample, and the average value was calculated.

We determined the tensile impact strength of the samples at 23 °C using a tensile impact tester (No.511; MYS-TESTER Co., Ltd., Osaka, Japan) according to method A of JIS K 7160. The films (0.5 mm thick) were punched to produce test specimens of shape 3, as specified in JIS K 7160. The measurements were obtained five times for each sample, and the average value was calculated with its standard deviation. After the impact tests, the broken pieces were used for investigation by transmission electron microscopy (TEM) (H7650; Hitachi High-Tech Corp., Tokyo, Japan) at an accelerating voltage of 100 kV. The fractured area was sliced to a thickness of 100 nm using an ultramicrotome (FC6; Leica Microsystems GmbH) at −90 °C and stained with ruthenium tetroxide vapor.

The biodegradability of each sample in seawater was evaluated by biochemical oxygen demand (BOD) tests. The seawater was collected from Takasago Port (Hyogo, Japan) and screened using an 80 µm mesh before use. Each sample weighing 25 mg was placed in 250 mL of seawater to which 0.05 g L^−1^ of ammonium chloride and 0.1 g L^−1^ of potassium dihydrogen phosphate were added. The seawater containing the sample was continuously stirred at 30 °C, and BOD data were collected daily. The BOD values were determined by measuring the pressure loss arising from the absorption of the CO_2_ generated by microbial respiration by KOH tablets with a BOD measuring device (Oxitop; Xylem Analytics Germany Sales GmbH & Co. KG, Weilheim, Germany) capable of using a pressure sensor to measure the pressure drop of a gas phase in an airtight container under constant temperature conditions. The biodegradability was calculated using the following equation:

Biodegradability = (BOD_t_ − BOD_b_)/ThOD × 100 (%) (2)

where BOD_t_, BOD_b_, and ThOD represent the BOD of the test solution, the BOD of the control blank, and the theoretical oxygen demand (the amount of oxygen required for complete decomposition into water and carbon dioxide), respectively.

## 3. Results and Discussion

### 3.1. Structure of Blends

**Figure 1:**
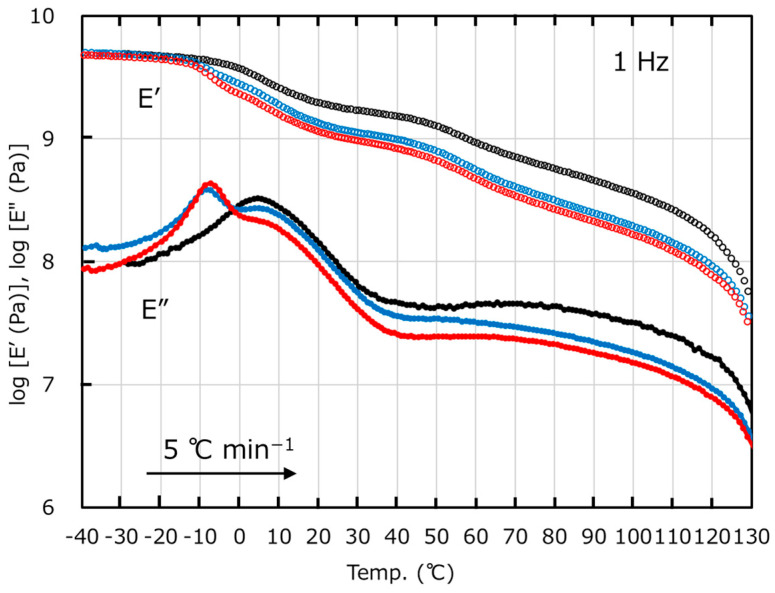
Temperature dependence of the tensile storage modulus *E*′ (open symbols) and loss modulus *E*″ (closed symbols) at 1 Hz of the compression-molded films of PHBH6 (black), PHBH6/uPHBH28 (70/30) (blue), and PHBH6/xPHBH28 (70/30) (red).

Figure 1 shows the temperature dependence of the dynamic tensile moduli, i.e., the storage modulus *E*′ and loss modulus *E*″ at 1 Hz of PHBH6, PHBH6/uPHBH28 (70/30), and PHBH6/xPHBH28 (70/30). PHBH6 exhibited the typical viscoelastic behavior of a crystalline polymer. There was a distinct peak at 4.9 °C in the *E*″ curve that was attributable to the glass-to-rubber transition, i.e., *T_g_*. Correspondingly, *E*′ decreased in this temperature region. The result corresponded with the previous report [36]. Furthermore, a broad peak due to α-dispersion was detected in the temperature range from 60 to 110 °C, which is a mechanical relaxation of amorphous chains confined by the crystalline phase [37]. The blends had lower *E*′ values in the wide temperature range, which was obvious in the blend with xPHBH28. Furthermore, there were two *E*″ peaks corresponding to *T_g_* of both phases, demonstrating phase separation. The peak temperatures due to *T_g_* of the dispersions occurred at −8.5 °C in PHBH6/uPHBH28 and −7.4 °C in PHBH6/xPHBH28. They were lower than that of PHBH6. This is reasonable because *T_g_* of PHBH decreases with increasing the 3HHx content [19]. Moreover, *T_g_* is known to increase by crosslinking because the segmental motion is restricted [38]. Although the peak temperature, *T_g_*, of PHBH6 was not affected by the addition of the other component, the peak intensity in the blend with crosslinked particles, which occurred at approximately 5 °C, was weak. This weak *E*″ peak of PHBH6 and the low *E*′ values beyond *T_g_* of PHBH6/xPHBH28 (70/30) will be discussed later with the morphology observations.

**Figure 2:**
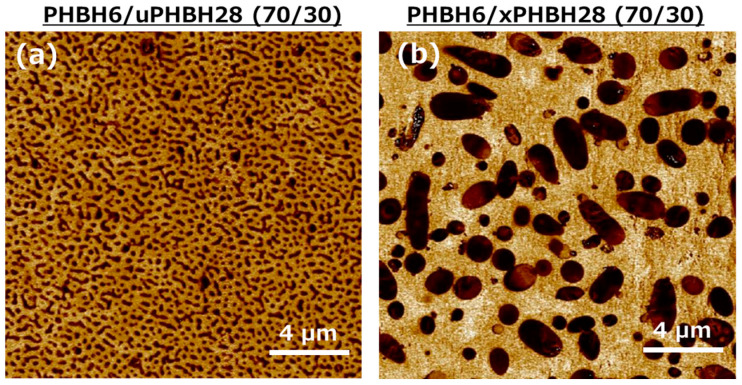
SPM modulus mapping images at the cut surfaces of the extruded strands: (**a**) PHBH6/uPHBH28 (70/30) and (**b**) PHBH6/xPHBH28 (70/30). SPM = scanning probe microscopy.

Figure 2 shows SPM images obtained at the centers of the extruded strands. In the figure, the dark areas represent a low modulus, i.e., PHBH28. In both samples, the phase-separated structure was clear. The particles of xPHBH28 were approximately 1.7 µm in diameter, which was the same as that of the original particles before mixing. This suggests that the particles did not break/disintegrate during melt-mixing because of their crosslinked structure. In other words, the particle size in the blends can be controlled irrespective of the blend ratio. In contrast, PHBH6/uPHBH28 (70/30) had a fine structure, in which the uPHBH28 particles were smaller than 0.5 µm. This result demonstrates the favorable mixing performance of the compounder.

**Figure 3:**
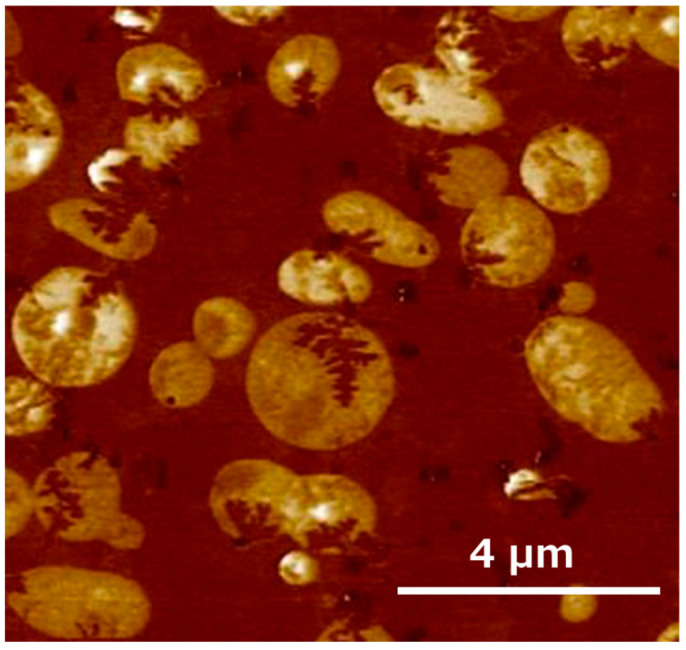
SPM adhesion mapping image at the cut surface of PHBH6/xPHBH28 (70/30). SPM = scanning probe microscopy.

The SPM adhesion mapping image shown in Figure 3 reveals the presence of crystalline structures inside some of the xPHBH28 particles. Because PHBH28 is an amorphous polymer, this indicates that some PHBH6 polymer chains were incorporated into the particles and crystallized. We further evaluated the ratio of the dark area from the modulus mapping. The area ratios of the continuous phase were 66.4% for PHBH6/uPHBH28 (70/30) and 63.3% for PHBH6/xPHBH28 (70/30). This observation also suggests the incorporation of PHBH6 chains into xPHBH28. The decrease in the continuous phase must be responsible for the low *E*′ values beyond *T_g_*. Although the exact incorporation mechanism is unclear at present, this phenomenon was detected in the blends with crosslinked particles when mixed under elongational flow [39,40,41]. During flow in the mixer, a negative pressure is generated in deformed particles with a crosslinked structure. This is responsible for the incorporation of polymer chains in the continuous phase.

**Figure 4:**
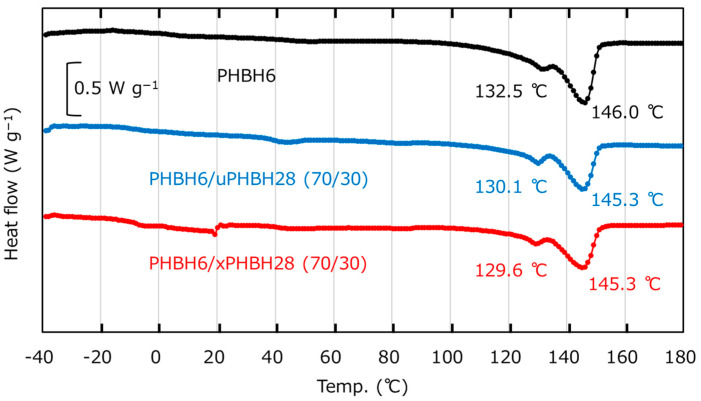
DSC heating curves obtained at 10 °C min^−1^: PHBH6 (black), PHBH6/uPHBH28 (70/30) (blue), and PHBH6/xPHBH28 (70/30) (red). DSC = differential scanning calorimetry.

DSC heating curves are shown in Figure 4. The melting points (*T_m_*’s) of PHBH6 appeared at 132.5 °C (*T_m_*_1_) and 146.0 °C (*T_m_*_2_). The peak at *T_m_*_1_ can be ascribed to small crystals generated during annealing at 23 °C after compression molding, whereas *T_m_*_2_ is due to the melting of the crystals formed during heating when the measurements were obtained [42]. Both *T_m_*_1_ and *T_m_*_2_ were barely affected by the addition of PHBH28, irrespective of the crosslinking procedure. This was as expected because of their phase-separated structure. The heat of fusion (*Δh_F_*) values were determined to estimate the crystallinity and were as follows: 38.5 J g^−1^ for PHBH6/uPHBH28 (70/30) and 38.6 J g^−1^ for PHBH6/xPHBH28 (70/30). The crystallinity calculated using Equation (1) was approximately 26.4% for both blends.

### 3.2. Mechanical Properties

**Figure 5:**
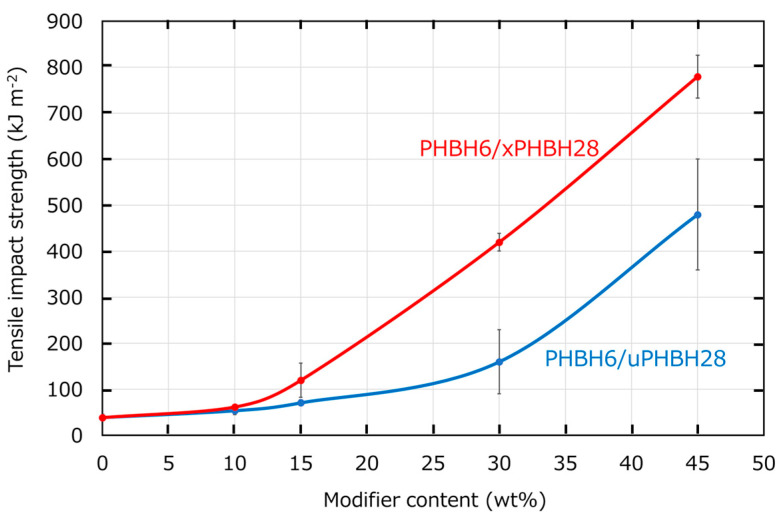
Tensile impact strength of the samples with various amounts of uPHBH28 (blue) or xPHBH28 (red).

The tensile properties are summarized in Table 1. As expected, the addition of the modifier decreased the tensile modulus and yield strength owing to the decrease in the high-modulus component, i.e., PHBH6. The low-tensile modulus of the blends with xPHBH28 compared with those with uPHBH28 corresponded to the tensile storage modulus *E*′ at 23 °C (Figure 1). Moreover, the blends with xPHBH28 achieved higher elongation at break than those with uPHBH28, which was pronounced in the blends with 30 and 45 wt% modifiers. In these blend ratios, the fracture stresses of the blends with xPHBH28 were higher than those with uPHBH28. Consequently, the blends with xPHBH28 had greater mechanical toughness than that with uPHBH28. These results corresponded well with the higher tensile impact strength described in Figure 5.

The tensile impact strengths of the samples with various amounts of uPHBH28 and xPHBH28 are presented in Figure 5. The tensile impact strength was enhanced by the addition of uPHBH28 and xPHBH28. Furthermore, xPHBH28 exhibited a superior modification performance compared with uPHBH28. In the case of xPHBH28, a notable increase in the impact strength was detected at/beyond 15 wt%. Although a simple comparison is not allowed, the modification performance of xPHBH28 was more significant than those of the other biodegradable soft materials, such as PCL [21], PBSA [22], and PBAT [23].

**Figure 6:**
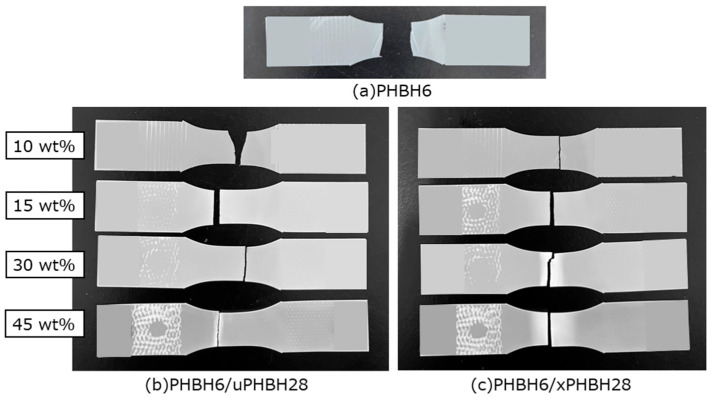
Specimens of (**a**) PHBH6, (**b**) PHBH6/uPHBH28 blends, and (**c**) PHBH6/xPHBH28 blends after the tensile impact tests.

Figure 6 shows the specimens after the tensile impact tests. The PHBH6 specimen was broken into several small pieces by the impact. Except for PHBH6/uPHBH28 (90/10), the others were broken into two pieces. This suggests that the fracture mechanism was changed by the modifier. Moreover, stress-whitening was apparent around the fractured areas in the samples with 30 and 45 wt% of xPHBH28. In the blends with uPHBH28, stress-whitening was detected only in the restricted area near the fractured surface, when the content was 45 wt%. These fracture mechanisms corresponded well with the impact strength, i.e., the blends with xPHBH28 had higher impact strength. This was as expected because void opening, i.e., the origin of stress-whitening, decreases the dilatational stress [37,43,44,45,46].

**Figure 7:**
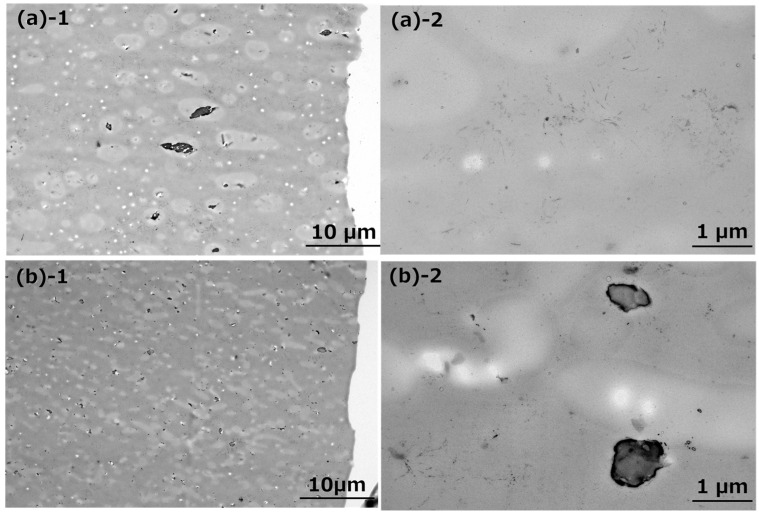
TEM images of thin slices of the fractured area of (**a**) PHBH6/uPHBH28 (70/30) and (**b**) PHBH6/xPHBH28 (70/30) after tensile impact tests: (**1**) ×5000 and (**2**) ×40,000. TEM = transmission electron microscopy.

To clarify the impact resistance mechanism, we carried out TEM investigations of the fractured samples of PHBH6/uPHBH28 (70/30) and PHBH6/xPHBH28 (70/30). In Figure 7, the dark and gray areas represent PHBH6 and PHBH28, respectively, and the white ones are voids.

First, the dispersed size of uPHBH28 was significantly different from that after the extrusion shown in Figure 2a. This demonstrated that the droplets coalesced during compression molding. In contrast, xPHBH28 was almost the same size after extrusion. Consequently, the particles in the uPHBH28 dispersions were much larger than the xPHBH28 particles. This characteristic is one of the advantages of crosslinked particles in terms of the stabilization of the dispersed rubber size. In the case of the blends with uPHBH28, the size of the dispersed droplets increased with the blend ratio because droplets frequently coalesce at high contents [47]. Moreover, these results indicate that the mechanical properties of the uPHBH28 blends must have depended on the processing method, i.e., the type of extruder, the screw geometry, the screw rotation speed, the resin temperature, and the residence time. During conventional processing operations, the structure is usually not in the equilibrium state. Consequently, there is a large distribution of the droplet size. In fact, the standard deviation of the blend with uPHBH28 was larger than that with xPHBH28 during the tensile impact test (Figure 5). Because of the fine dispersion, the ligament thickness in the blend with xPHBH28 was smaller than that in the blend with uPHBH28. Such a situation leads to shear yielding, i.e., ductile deformation [26]. This would explain the large elongation at break during the tensile tests and the high mechanical toughness during the impact tests.

Second, spherical voids were detected in both samples. Such voids are always deformed in the stretching direction when generated. After the impact test, they shrank to spheres owing to elastic recovery, because segmental motion is allowed at room temperature, i.e., beyond *T_g_*. In fact, our results verified that the deformed PHBH exhibited rubber-like mechanical responses [48]. Considering the intense stress-whitening in the blend with xPHBH28, the initial voids before elastic recovery must have been larger. Furthermore, the location of the voids differed between the two samples (PHBH6/uPHBH28 and PHBH6/xPHBH28). Most of the voids detected in the blend with uPHBH28 were in the continuous phase and must have formed from crazing in PHBH6. In the case of the blend with xPHBH28, clear cavitations were detected in the rubber particles, as shown in Figure 7b-2. Considering that the cohesive strength must have been almost the same in uPHBH28 and xPHBH28, the frequent cavitation in xPHBH28 can be attributed to a large deformation during impact testing, as in the tensile tests. Cavitation is known to decrease the dilatational stress in the system, leading to favorable mechanical toughness [28,29]. The difference from the blend with uPHBH28 was probably due to the size effect. The small dispersion size of xPHBH28 was responsible for the shear yielding due to small ligament thickness, leading to large deformation, i.e., enhanced fracture energy. These things considered, cavitation must be pronounced greatly due to the large deformation, which contributed to the significantly high impact strength.

### 3.3. Biodegradability

**Figure 8:**
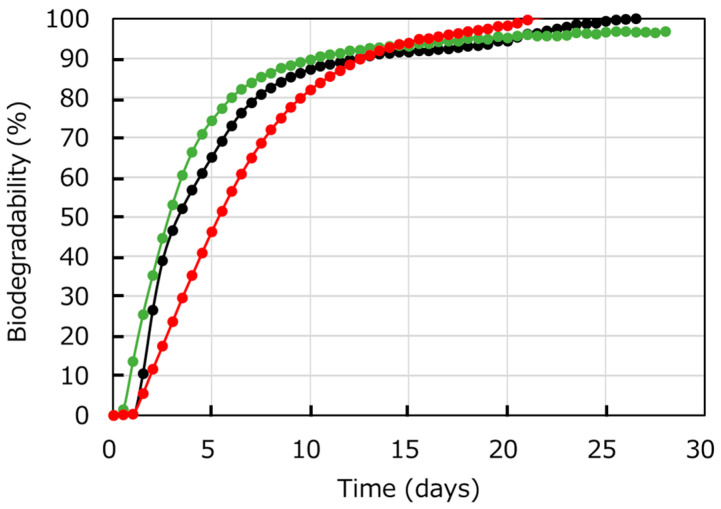
Biodegradability measured by BOD tests in seawater. PHBH6 (black), xPHBH28 (green), and PHBH6/xPHBH28 (70/30) (red). BOD = biochemical oxygen demand.

Figure 8 represents the biodegradability percentages of PHBH6, xPHBH28, and PHBH6/xPHBH28 (70/30) in seawater. Although the result depends on the composition of seawater, including the concentration of various ions, the BOD tests must be important to comprehend the biodegradability. As seen in the figure, each sample had excellent biodegradability in seawater. Over 90% biodegradability was achieved after only 2 weeks, and decomposition was complete after 4 weeks. Even though there was a difference in the initial rise, it must be owing to the biological nature of the method and therefore most likely within the range of experimental variation. Normally, degradation proceeds mainly in the amorphous phase, and lower crystallinity leads to a higher degradation rate owing to high water absorption [49,50]. Therefore, the favorable biodegradability of PHBH28 was as expected.

## 4. Conclusions

In the present study, we investigated the structure and properties of PHBH6 modified by uPHBH28 or xPHBH28. We found that the size of the dispersed uPHBH28 particles was sensitive to the processing method. In contrast, the xPHBH28 particles retained their form under any processing conditions, ensuring favorable and stable mechanical performance. It should be noted that small amounts of xPHBH28 achieved superior tensile impact strength compared with the amount of uPHBH28 required. This indicates that the stiffness of a PHBH blend can be maintained at a higher level, i.e., the mechanical balance between rigidity and impact strength is improved using xPHBH28. The superior modification performance of xPHBH28 over uPHBH28 is probably owing to its fine dispersion in PHBH6. Consequently, shear yielding occurs. Furthermore, a large deformation also induces cavitation in the rubber particles, which are the origin of stress-whitening and contribute to the mechanical toughness. Moreover, xPHBH28 itself, and the PHBH6/xPHBH28 blends had excellent biodegradability in seawater. Considering these findings, we conclude that the addition of xPHBH28 is one of the best ways of modifying the mechanical properties of PHA whilst retaining its favorable properties. This will expand its usefulness in various applications.

## Figures and Tables

**Table 1:** Tensile properties of the samples with various amounts of uPHBH28 or xPHBH28.

Sample	Tensile Modulus (MPa)	Yield Stress (MPa)	Fracture Stress (MPa)	Elongation at Break (%)
PHBH6	3030	33	32	2
PHBH6/uPHBH28 (90/10)	2460	28	20	17
PHBH6/uPHBH28 (85/15)	2130	27	16	21
PHBH6/uPHBH28 (70/30)	1590	16	15	100
PHBH6/uPHBH28 (55/45)	1110	12	14	125
PHBH6/xPHBH28 (90/10)	2260	26	18	13
PHBH6/xPHBH28 (85/15)	2120	26	17	22
PHBH6/xPHBH28 (70/30)	1580	18	18	140
PHBH6/xPHBH28 (55/45)	960	11	17	200

## Data Availability

Not available.
